# Heat-Induced Secretion of Heat Shock Proteins 70 and 90 Does not Affect the Expression of the Glucocorticoid Receptor in Primary Airway Cells in COPD

**DOI:** 10.1007/s00408-024-00680-8

**Published:** 2024-04-19

**Authors:** Liang Zhou, Lei Fang, Michael Roth, Eleni Papakonstantinou, Michael Tamm, Daiana Stolz

**Affiliations:** 1 Department of Clinical Research, University Hospital Basel and University of Basel, CH-4031 Basel, Switzerland; 2 Clinic of Respiratory Medicine and Pulmonary Cell Research, University Hospital Basel, CH-4031 Basel, Switzerland; 3 Clinic of Respiratory Medicine, Medical Center-University of Freiburg, 79106 Freiburg, Germany; 4 Faculty of Medicine, University of Freiburg, 79106 Freiburg, Germany

**Keywords:** Chronic obstructive pulmonary disease, Heat shock proteins, Glucocorticoid sensitivity, Glucocorticoid receptor, Bronchial epithelial cells, Airway smooth muscle cells, Toll-like receptor

## Abstract

**Purpose:**

The response to glucocorticoids is hampered in many COPD patients by a yet unknown mechanism. Earlier we reported that short-term heat exposure of primary human bronchial epithelial cells (BEC) and airway smooth muscle cells (ASMC) of asthma patients increased the expression and secretion of extracellular heat shock proteins (eHSPs) resulting in increased expression of glucocorticoid receptor (GR) in BEC and inhibition of ASMC remodeling. The aim of the present study was to assess if the same mechanism is also present in primary airway wall cells of COPD patients.

**Methods:**

Primary BEC and ASMC were established from endobronchial biopsies obtained from COPD patients (*n* = 73), who participated in the HISTORIC study, an investigator-initiated and driven clinical trial. Secretion and protein expression of HSPs was assessed by ELISA and Western blotting. Expression of total GR, its isoforms GRα and GRβ and toll-like receptor 4 (TLR4) was determined by Western-blotting.

**Results:**

Short heat exposure (65 °C, 10 s) of BEC resulted in a significant increase of the secretion of eHSP70 and eHSP90, while the intracellular protein was not altered. Heat treatment or exposure to eHSP70 or eHSP90 had no effect on the expression of GR and GR-isoforms. However, eHSP70 and eHSP90 significantly reduced the expression of TLR4.

**Conclusions:**

The results of this study indicate that primary airway cells from COPD patients respond differently to heat exposure and extracellular HSP70 or HSP90 than cells from asthma patients regarding the expression of GR and this may explain the reduced response to glucocorticoids in patients with COPD.

*Trial Registration*: ISRCTN11017699

**Supplementary Information:**

The online version contains supplementary material available at 10.1007/s00408-024-00680-8.

## Introduction

Chronic obstructive pulmonary disease (COPD) is a clinical syndrome resulting from a complex, cumulative and dynamic gene-environment interaction over lifetime [1]. The disease cannot be cured, and only a limited symptom control can be achieved [2, 3]. Inhaled glucocorticoids (ICS) are an effective therapy to control inflammation in chronic inflammatory lung diseases such as asthma, while in contrast, the response of COPD patients to ICS is inconsistent [3, 4]. The complex formation of the glucocorticoid receptor (GR) with heat shock proteins (HSPs) controls its function as an anti-inflammatory transcription factor and is of interest to investigate if in COPD this is associated with reduced response to ICS [5, 6].

The GR belongs to a protein superfamily of highly conserved steroid-hormone-activated transcription factors [7]. The GR encoding gene was assigned as nuclear receptor subfamily 3 group C member 1 (NR3C1) and can be transcribed into five isoforms: GRα, GRβ, GR-P, GRγ, and GR-A by alternative mRNA splicing or translation [8]. However, the clinically beneficial effects of ICS are mediated mainly through the GRα isoform and the presence of other GR-isoforms have been reported to alter its capacity of ligand-binding or GR-DNA interaction, and thereby modifies the cell’s response to ICS [9, 10].

HSPs are a family of highly conserved and ubiquitous molecular chaperone proteins across all species [11]. In cell, HSPs are essential for proteome maintenance such as protein folding, assembly of multiprotein complexes, and the transport of proteins into subcellular compartments [11, 12]. Intracellular (i)HSP70 and iHSP90 share targets including the GR and thus, regulate its signaling [13]. The complex formation between both iHSPs and GR is ATP-dependent and regulates the transport of the GR from the cytoplasm into the nucleus where it acts as a transcription factor [5]. iHSP70 keeps the GR in a ligand-binding conformation and is replaced by iHSP90 after ligand binding [5]. iHSP90 enables the GR to bind with a specific DNA sequence, named the glucocorticoid response element (GRE), which can either activate or repress gene transcription [7].

In asthma patients the level of secreted extracellular (e)HSP70 and eHSP90 was increased in bronchoalveolar lavage fluid (BALF) after bronchial thermoplasty [14]. Experimental heat therapy (65 °C for 10 s) also significantly increased the secretion of both HSPs by isolated bronchial epithelial cells (BEC). In contrast, heat treatment reduced the expression of both HSPs by airway smooth muscle cells (ASMC) from healthy controls and asthma patients [14]. Furthermore, heat significantly increased the expression and translocation of the GR by BEC of asthma and control groups [15]. Secreted HSP70, but not HSP90, increased the GR expression and in consequence that of the signaling inhibitor DUSP1 by BECs, thereby reducing the activity of pro-inflammatory signaling cascades [16]. In contrast, increased iHSP70 decreased GR expression by BEC [16].

This study aimed to investigate if the above-described mechanism of heat, HSP70 and HSP90 on the regulation of the GR and its isoforms, that was earlier described by us in asthma cells, also occurs in primary airway cells of COPD patients.

## Materials and Methods

### Patient Cohort

BEC and ASMC were isolated from endobronchial biopsies obtained from COPD patients (*n* = 73) in the Pneumology Clinic, University Hospital Basel, Switzerland. All patients participated in the HISTORIC study [17], an investigator-initiated and -driven, double-blind, randomized, placebo-controlled trial performed at the University Hospital of Basel, Switzerland. The study was approved by the Institution Review Board (EKNZ 2016–6-01880) and was registered with ISRCTN registry (ISRCTN11017699) [17] and proved the hypothesis that COPD patients with high ASMC area in their endobronchial biopsies (> 20% of the total biopsy area) respond better to ICS than patients with low ASMC area (≤ 20% of the total biopsy area). In the HISTORIC study, 190 COPD patients, Global Initiative for Chronic Obstructive Lung Disease Stage B–D, underwent bronchoscopy with endobronchial biopsy. Patients were divided into groups A and B, with high ASMC area (> 20% of the bronchial tissue area) and low ASMC area (≤ 20% of the bronchial tissue area), respectively, and followed a run-in period of 6 weeks on open-label triple inhaled therapy with aclidinium (ACL)/formoterol (FOR)/budesonide (BUD) (400/12/400 μg twice daily). Subsequently, patients were randomised to receive either ACL/FOR/BUD or ACL/FOR/placebo and followed for 12 months. The primary endpoint of the study was the difference in post-bronchodilator forced expiratory volume in 1 s (FEV1) over 12 months between patients with LASMC and HASMC receiving or not receiving ICS. At the end of the study patients who were receiving ACL/FOR/BUD were characterized as responders if they had an improvement if FEV1 or as non-responders if they had a decline in FEV1.

### Primary Cultures of BEC and ASMC

Primary BECs and ASMC were established from endobronchial biopsies of COPD patients (*n* = 73) obtained at the Department of Pneumology, University Hospital Basel, Switzerland. Among these COPD patients 25 belonged to the group of high ASMC area and 48 patients belonged to the group of low ASMC area, 13 patients responded to treatment with ICS (responders) and 27 patients did not respond to treatment with ICS (non-responders). Cells were grown in cell-type specific medium as described earlier [14].

Cells were exposed to recombinant human HSP70 (5 nM) and recombinant human HSP90 (5 nM) purchased from R&D Systems (Abingdon, UK) for 24 h.

Heat treatment was performed in confluent cells grown in T-25 flasks which were submerged into a water bath for 10 s, at 65 °C before new medium was added and cells further incubated under standard cell culture conditions over 24 h, as described earlier [14].

### Reagents and Antibodies

The antibodies used in this study are listed in Table 1.

**Table 1:** Primary and second antibodies list used for Western-blot

Primary antibody	Dilution	Catalog #	Company
GR	1:1000	ab183127	Abcam
HSP70	1:2000	#4873	Cell signaling
HSP90	1:1000	#4877	Cell signaling
GAPDH	1:1000	ab181602	Abcam
TLR4	1:1000	NB100-56579	Novus biologicals
Anti-Rabbit IgG	1:2000	A9169-2ML	Sigma

### Western Blotting

For Western-blots, cells were lysed in RIPA buffer (#SLCD5849, Sigma, USA) and the protein concentration of each sample was determined by BCA protein assay kit (#XI357440, ThermoFisher Scientific, Waltham, USA). The protein concentration was adjusted to 20 µg of total protein which were denatured (10 min., 95 °C), and applied to electrophoresis was for size fractionation (110 V, open Amp, 50 min., at 4 °C) in a 4–12% SDS–PAGE (#M41212, GeneScript, Piscataway, USA). Proteins were then transferred onto a nitro-cellulose membrane (#88018, ThermoFisher) by heat-accelerated capillary transfer and over-night incubation at 50 °C. Primary antibodies (Table 1) were applied overnight at 4 °C, followed by visualization with secondary, horse radish labeled antibodies. Blots were identified and quantified by imageJ (version 1.53).

### Enzyme-Linked Immunosorbent Assay (ELISA)

The secretion of HSP70 and HSP90 was determined by ELISA kit (HSP70: ADI-EKS-700B, HSP90: ADI-EKS-895, ENZO Life Science, Lausen, Switzerland), according to the manufacturer's instructions.

### Statistical Analysis

GraphPad Prism 9.0 software was used for data analysis. Data are represented as mean ± SEM. Statistical analysis was performed by Student’s t-test or one-way ANOVA test. The data were presented as mean ± SEM of the results from at least three independent experiments. *P*-value < 0.05 was considered statistically significant.

## Results

### Effect of Heat Exposure on the Secretion and Expression of HSP70 and HSP90 by Primary BEC

**Fig. 1:**
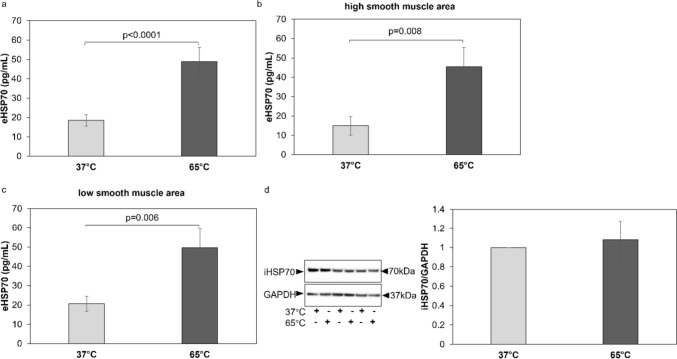
eHSP70 secretion and expression by primary isolated BEC from COPD patients exposed to 65 °C for 10 s. **a** eHSP70 secretion from primary COPD BEC (*n* = 54) before and after heat exposure (10 s 65 °C) measured by ELISA. **b**, **c**: eHSP70 secretion from primary COPD BEC from patients with high smooth muscle mass (*n* = 20) and low smooth muscle mass (*n* = 34), in their endobronchial biopsies. **d** Representative Western-blots and image quantification (*n* = 20) by image J. Βars represent fold change to cells cultured under 37 °C and show mean ± S.E.M. *P*-values were calculated by Student’s t-test

In BEC, heat treatment at 65 °C for 10 s, significantly increased the secretion of eHSP70 (*p* < 0.0001) (Fig. 1a). The significant increase of eHSP70 in response to heat was evident in BEC obtained from patients with high ASMC area (*p* = 0.008) (Fig. 1b) and from patients with low ASMC area (*p* = 0.006) (Fig. 1c). However, heat exposure of BEC did not affect protein expression of iHSP70 as assessed by western blotting (Fig. 1d).

**Fig. 2:**
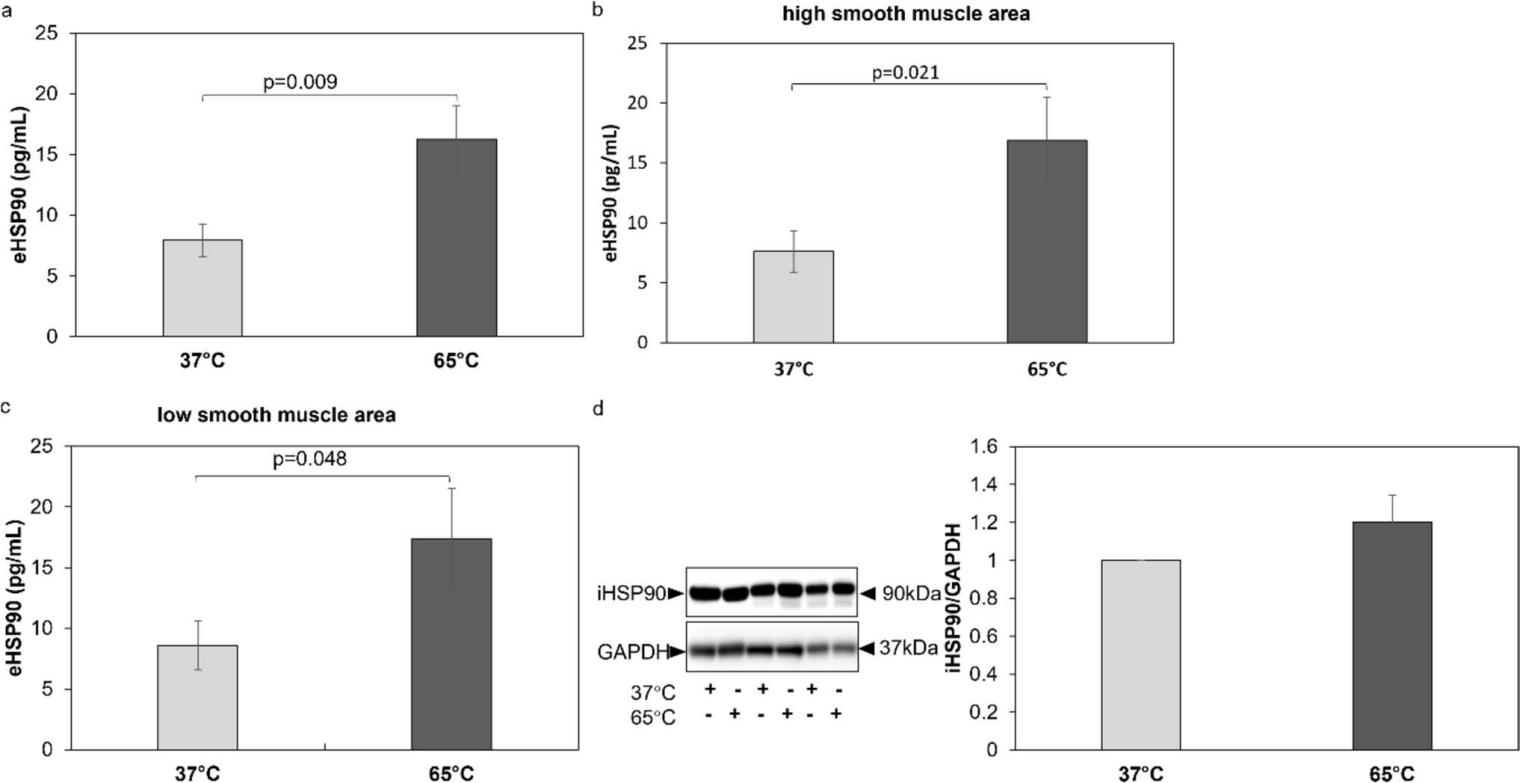
eHSP90 secretion and expression by primary isolated BEC from COPD patients exposed to 65 °C for 10 s. **a** eHSP90 secretion from primary COPD BEC (*n* = 54) before and after heat exposure (10 s 65 °C) measured by ELISA. **b**, **c**: eHSP70 secretion from primary COPD BEC from patients with high smooth muscle mass (*n* = 20) and low smooth muscle mass (*n* = 34), in their endobronchial biopsies. **d** Representative Western-blots and image quantification (*n* = 20) by image J. Βars represent fold change to cells cultured under 37 °C, and show mean ± S.E.M. *P*-values were calculated by Student’s t-test

Exposure of BEC at 65 °C for 10 s significantly increased the secretion of eHSP90 (*p* = 0.009) (Fig. 2a) and this was evident for BEC obtained from patients with high ASMC area (*p* = 0.021) (Fig. 2b) and from patients with low ASMC area (*p* = 0.048) (Fig. 2c). However, heat exposure of BEC did not affect protein expression of iHSP90 as assessed by western blotting (Fig. 2d). These results indicate that ASMC area in endobronchial biopsies does not affect the response of ASMC to heat regarding the expression of eHSP70, iHSP70, eHSP90 and iHSP90. Furthermore, when BEC were stratified according to respective patient’s response to ICS there was no significant difference in eHSP70 or eHSP90 in response to heat between responders and non-responders (Supplementary Fig. 1).

### Effect of Heat Exposure on the Expression of GR

**Fig. 3:**
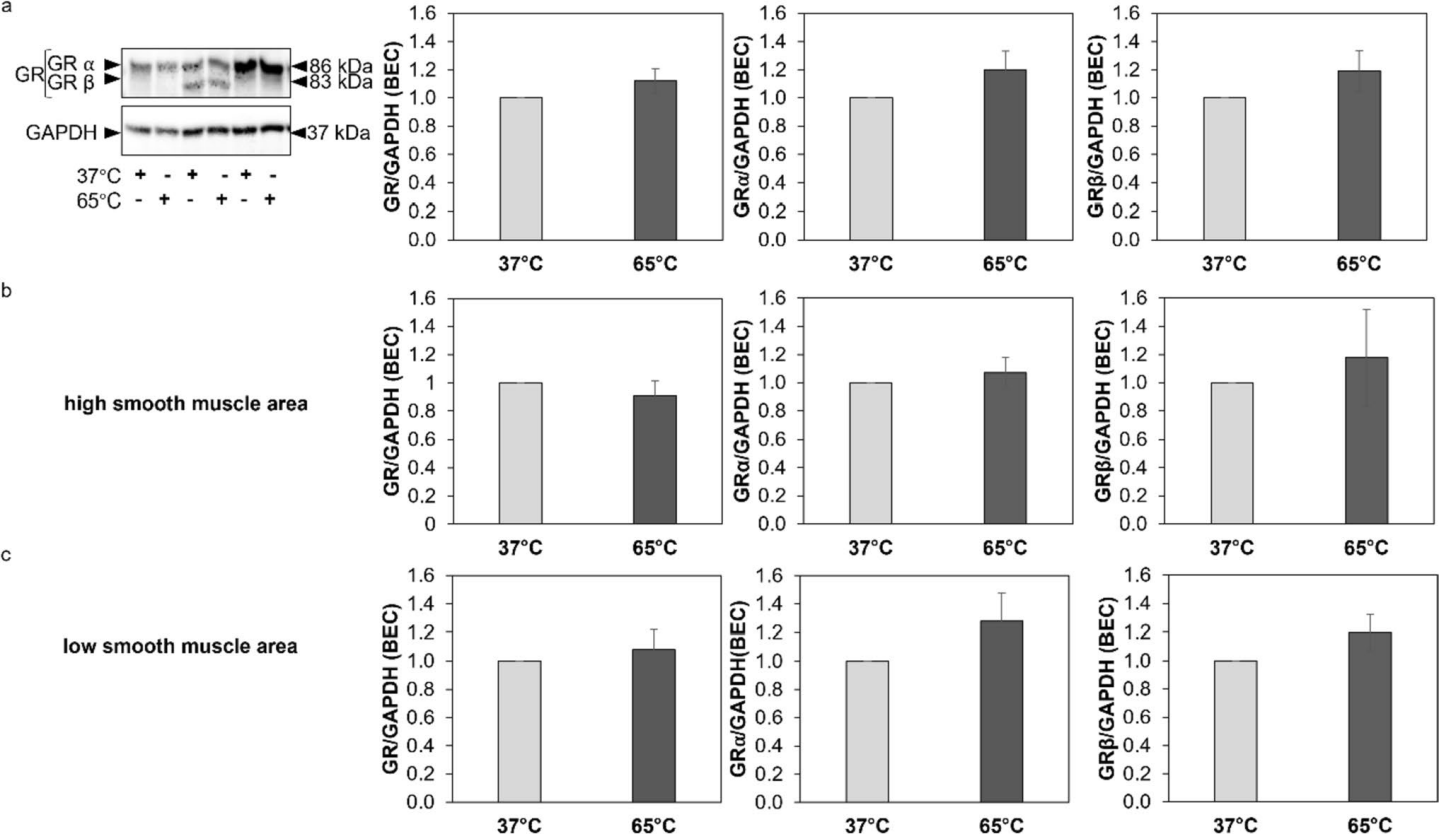
GR and GR-isoforms expression in primary BEC from COPD patients exposed to 65 °C for 10 s. **A** Representative Western-blots of the effect of heat on the expression of GR and GR-isoforms in primary BEC from COPD patients (*n* = 16) and quantitation of the western blots performed by Image J. **B**, **C**: Expression of GR and GR-isoforms in primary BEC from COPD patients with high smooth muscle mass (*n* = 6) and low smooth muscle mass (*n* = 10) in their endobronchial biopsies. Βars represent fold change of GR, GRα, and GRβ expression to cells cultured under 37 °C. Expression of total GR represents the sum of the expression of GRα and GRβ isoforms. Bars show mean ± S.E.M. *P*-values were calculated by Student’s t-test

Exposure of BEC at 65 °C for 10 s did not alter the expression of total GR or its isoforms GRα and GRβ as assessed by Western blotting (Fig. 3a). Similarly, short heat exposure did not affect the expression of GR or its isoforms GRα and GRβ in BEC when cells stratified according to high (Fig. 3b) or low (Fig. 3c) ASMC area in the respective endobronchial biopsies. Τhe ratio of GRα to GRβ was also not affected by heat in primary BEC (Supplementary Fig. 2a–c). Furthermore, when BEC were stratified according to respective patient’s response to ICS there was no significant difference in the expression of total GR, or its isoforms GRα and GRβ, or the ratio of GRα to GRβ in response to heat between responders and non-responders (Supplementary Fig. 3).

**Fig. 4:**
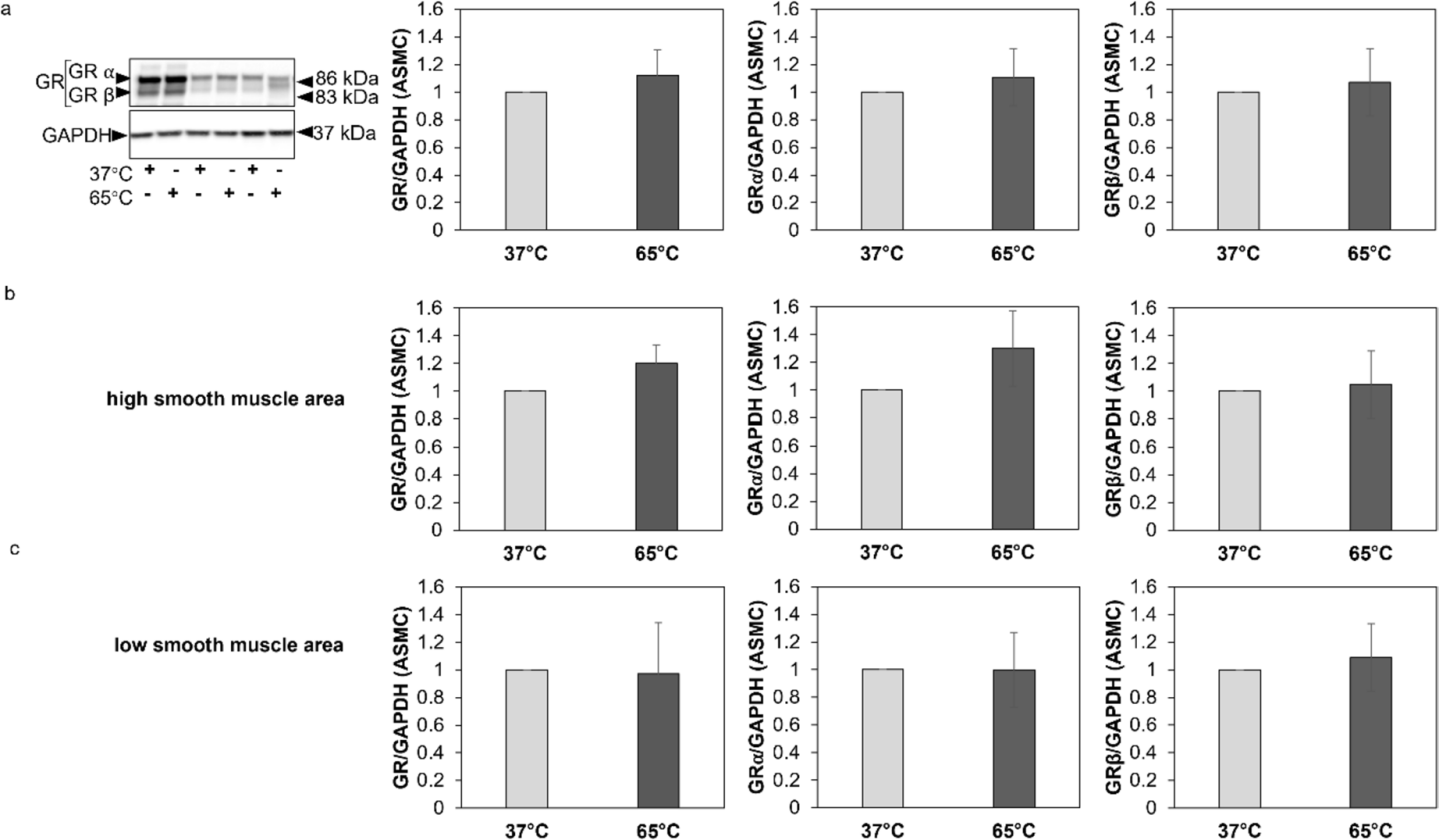
GR and GR-isoforms expression in primary ASMC from COPD exposed to 65 °C for 10 s. **a** Representative Western-blots of the effect of heat on the expression of GR and GR-isoforms in primary ASMC from COPD patients (*n* = 16) and quantitation of the western blots performed by Image J. **b**, **c**: Expression of GR and GR-isoforms in primary BEC from COPD patients with high smooth muscle mass (*n* = 6) and low smooth muscle mass (*n* = 10) in their endobronchial biopsies. Βars represent fold change of GR, GRα, and GRβ expression to cells cultured under 37 °C. Expression of total GR represents the sum of the expression of GRα and GRβ isoforms. Bar shows mean ± S.E.M. *P*-values were calculated by Student’s t-test

In ASMC, the expression of total GR and its isoforms GRα and GRβ did not change after exposure at 65 °C for 10 s (Fig. 4a). Likewise, when cells stratified according to high (Fig. 4b) or low (Fig. 4c) ASMC mass in the respective endobronchial biopsies, there was no difference in the expression of GR, GRα and GRβ in response to short heat exposure. Τhe ratio of GRα to GRβ was also not affected by heat in primary ASMC (Supplementary Fig. 4a-c). Furthermore, when ASMC were stratified according to respective patient’s response to ICS there was no significant difference in the expression of total GR, or its isoforms GRα and GRβ, or the ratio of GRα to GRβ in response to heat between responders and non-responders (Supplementary Fig. 5).

### Regulation of the Expression of GR in Primary ASMC by eHSPs

**Fig. 5:**
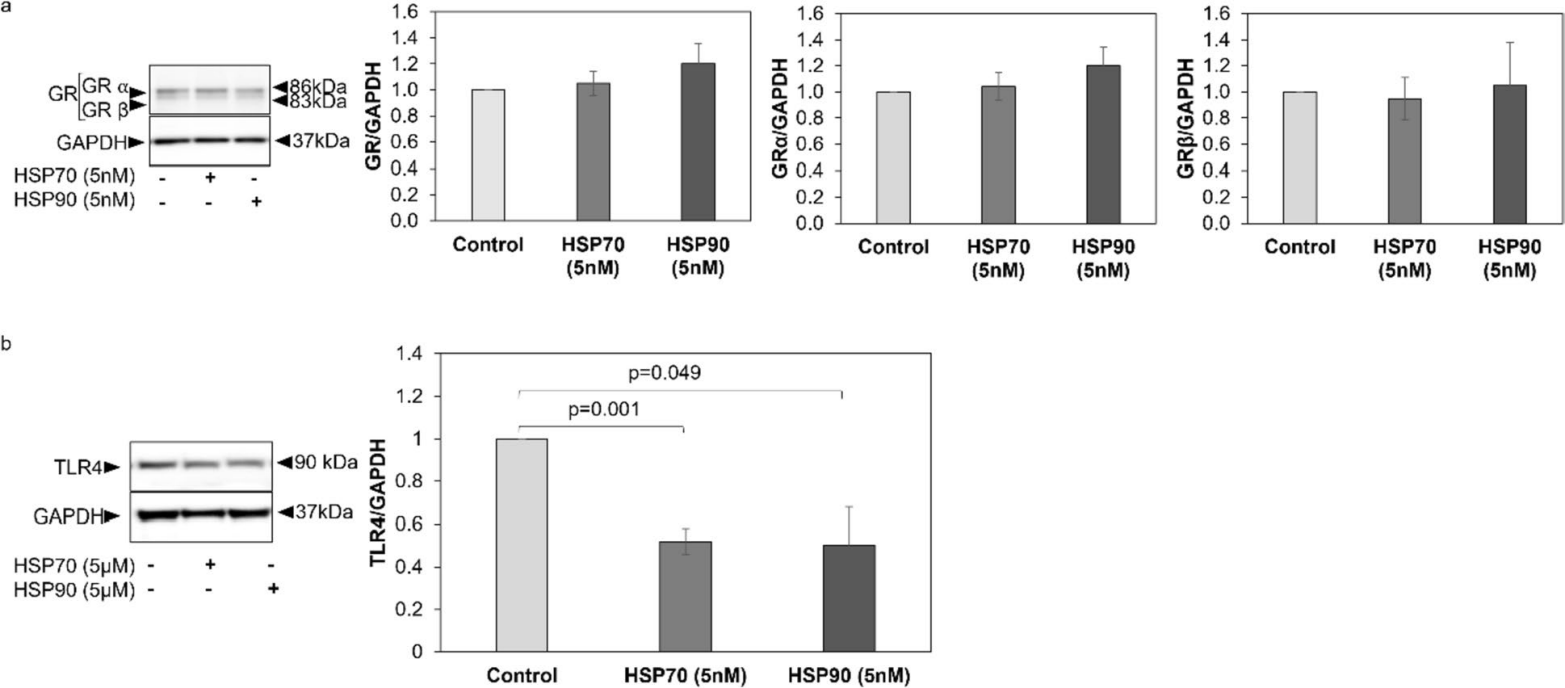
Effect of eHSPs on the expression of GR isoforms and TLR4. **a** Representative Western-blots for the effect of eHSPs on the expression of GR and GR-isoforms by ASMC from COPD patients (*n* = 7) and quantitation of the Western blots performed by Image J. Βars represent fold change of GR, GRα, and GRβ expression in exposed to eHSP70 (5 nM) or eHSP90 (5 nM) to cells without eHSPs tratment over 24 h. **b** Representative Western-blots for the effect of eHSPs on the expression of TLR4 in ASMC from COPD patients (*n* = 3) and quantitation of the Western blots performed by Image J. Βars represent fold change of TLR4 expression in ASMC (*n* = 3) exposed to eHSP70 (5 nM) or eHSP90 (5 nM) to cells without eHSPs treatment over 24 h. Expression of total GR represents the sum of the expression of GRα and GRβ isoforms. Bars show mean ± S.E.M. *P*-values were calculated by Student’s t-test or one-way ANOVA test

To assess the effect of eHSPs on the expression of GR in primary ASMC from COPD patients, cells were exposed to recombinant human HSP70 (5 nM) or recombinant human HSP90 (5 nM) for 24 h. Νeither eHSP70 nor eHSP90 had a significant effect on the expression level of GRα or GRβ (Fig. 5a) or in the ratio of GRα/GRβ (Supplementary Fig. 6).

In a small sample of COPD-derived primary ASMC (*n* = 3), we could demonstrate that the expression of TLR4 was significantly reduced after exposure to either eHSP70 (*p* = 0.001) or eHSP90 (*p* = 0.049) (Fig. 5b).

## Discussion

Earlier we reported that short-term heat exposure of primary human BEC and ASMC of asthma patients increased the expression and secretion of eHSPs, which increased the expression of the glucocorticoid receptor (GR) in BEC and inhibited remodeling of ASMC [14, 16]. The aim of the present study was to assess if the same mechanism is also present in BEC of COPD patients. The results of this study indicate that BEC and ASMC from COPD patients respond differently to heat exposure than cells from asthma patients regarding the expression of GR. This finding may explain the reduced response to glucocorticoids in patients with COPD.

Asthma and COPD are both most common chronic inflammatory respiratory diseases affecting 1 in 12 people worldwide [2, 18]. In patients with COPD, pathological changes appear in the airways, the lung parenchyma, and the pulmonary vasculature [19]. COPD is characterized by neutrophilic inflammation [20]. Neutrophils, together with BEC and other structural cells such as ASMC, release a multitude of inflammatory mediators, which leads to persistent airflow remodeling and sub-sequent airway obstruction [2]. Thus, the inflammation in COPD is different from the inflammatory pattern in asthma, which is mainly due to eosinophilic inflammation [20], leading to bronchial hyperresponsiveness [21].

In asthmatic BEC, iHSP70 and iHSP90 were up-regulated after heat treatment in vitro and in vivo whereas both iHSPs were down-regulated in asthmatic ASMC in vitro and in vivo [14]. Heat treatment increased eHSP70 and eHSP90 secretion by asthmatic BEC but did not induce eHSP70 secretion by asthmatic ASMC [14]. In this study, we observed an increase of secreted eHSP70 and eHSP90 by isolated COPD BEC after heat treatment, while no significant change of iHSP70 and iHSP90 expression was observed, indicating a different effect of heat exposure in BEC in asthma and COPD. Therefore, the export of iHSP70 and 90 into the extracellular environment in isolated BEC from COPD patients is needed to be further investigated. In this study, no change of eHSP70 and eHSP90 by heat between responders and non-responders suggests that eHSPs level and their change by heat could not be used for prediction of response to ICS in vitro*.* This is in line with the study that no association observed between glucocorticoid response and HSP90 in vivo [22]. Human recombinant HSP70 (rhHSP70) regulated iHsp70 expression via TLR4 in macrophages [23], which was also a receptor for eHSP90 in macrophages [24]. In this study, we observed a reduced expression of TLR4 after exposure to eHSP70 (5 nM). Thus, the reduction of TLR4 by eHSP70 might explain the difference of iHSP70 and iHSP90 in BEC from between COPD and asthma patients.

Regarding the observed reduce expression TLR4 after treatment of cells with HSP70 or HS90, it can be assumed that this results from complex formation between both HSPs with TLR4 and sub-sequent internalization, which had been reported in monocyte-derived dendritic cells by others [25]. It is indicated that TLR4 internalization is a common mechanism that follows complex formation with ligands such as lipopolysaccharides (LPS) or GPR78 and leads to reduced TLR4 expression [26, 27]. Reduced TLR4 expression might affect the immune response of COPD patients to infection, as bacterial LPS bind and activate the TLR4 [28]. In this context it is of interest to note that COPD patients have increased levels of circulating HSP70 [29, 30]. These findings might explain the reduced response to inhaled steroids by down-regulation of TLR4, that we report here. On the other hand, it might be the basis of the increased inflammation as consequence of TLR4 activation [31]. Further studies using endobronchial tissue biopsies from patients with COPD are needed to confirm the in vitro data presented in this study and to elucidate under which clinical conditions the anti-inflammatory effect of eHSPs will counterbalance the pro-inflammatory effect of TLR4. Other studies, however, indicated that the interaction between LPS and TLR4 led to an increased sensitivity to steroids in cell and animal models [32, 33]. The different effects of TLR4 on the regulation of GR imply that assessment of such mechanisms has to carefully consider cell- or species-specific signaling sequences. In an earlier study we reported that, heat increased eHSP70 and subsequently GR expression as well as its activation in control and asthma BEC [15]. However, in BEC and ASMC from COPD patients, neither heat nor treatment with eHSPs affected the expression of the GR isoforms. Stolz et al. [17] found that in COPD patients the smooth muscle mass might correlate with ICS responsiveness. The data presented in this study, did not indicate any difference of GR isoforms expression between COPD patients with high and low smooth muscle mass. Therefore, expression of GR and GR isoforms could not be used to indicate the individual response to ICS. It was also reported failure to use GR-isoform ratios to predict patient’s response to steroid in young adults [34].

Increased iHSP70 expression negatively correlated with FEV1 and GR expression in COPD [16, 35]. Furthermore, high expression of iHSP70 was associated with production of excess mucus, which is a feature of COPD [36, 37]. Treatment with eHSP70 had anti-inflammatory effects through regulation of GR and increased BEC proliferation but decreased proliferation and differentiation in ASMC [14, 16]. The stimulatory effect of eHSP70 on GR expression in asthma and control BEC was mediated by TLR4 [16]. As shown here, eHSP70 had no such effect in isolated BEC from patients with COPD, which might be due to the downregulation of TLR4 by eHSP70. The mechanisms involved in this negative feedback of TLR4 by eHSP70 should be further investigated. In this respect, it would be of interest to study the expression of Rab7B which has been shown to be induced in COPD epithelia [38] and is known to lead to TLR4 degradation [39] and to compare the expression of Rab7D in COPD and asthmatic epithelia. Furthermore, the differential expression of Dectin-1 which is a receptor expressed in human lung [40] and has been shown that it suppresses TLR4 expression [40] should also be investigated in COPD and asthmatic epithelia. Future studies have to investigate the possibility of a cell-type specific effect of eHSPs as reported by others [41]. Furthermore, when assessing the effects of eHSP70 in the context of COPD the presence of inhibitory acting HSP auto-antibodies, which have been reported in COPD patient’s sputum and serum [42], has to be taken into account. Thus, it will be necessary to determine the ratio of free and antibody bound HSPs in COPD patient; such data are currently missing.

A significant limitation of our study is the short life span and limited expansion of primary human BECs, which hindered obtaining a sufficient number of cells for an in-depth investigation into the exact mechanisms underlying the reduced expression of TLR4 in response to HSP70 and HSP90. This constraint also prevented further experiments to explore whether the interaction of TLR4 with the GR might be cell-type or organ-specific [43], or if TLR4 and GR interact with the same chaperone proteins such as HSPs to regulate basic cell functions, and whether silencing either molecule may impact the function of the other [33]. However, a strength of our study is that it is the first to involve primary BEC and ASMC from a well-characterized COPD cohort, distinguishing between responders and non-responders to ICS, based on a randomized, placebo-controlled clinical trial.

In conclusion, our data suggest that in COPD, neither heat treatment nor eHSP70 supported ICS signaling by up-regulating the GR. The observation that eHSPs downregulated the expression of their shared receptor TLR4 might explain the loss of mechanism on GR expression in COPD and needs to be further investigated.

### Supplementary Information

Below is the link to the electronic supplementary material.

Supplementary file1 (DOCX 585 kb)

## Data Availability

All original data can be requested from the first author.

## Abbreviations

ASMC: Airway smooth muscle cell
BALF: Bronchoalveolar lavage fluid
BEC: Bronchial epithelial cell
COPD: Chronic obstructive pulmonary disease
DUSP-1: Dual specificity phosphatase 1
GR: Glucocorticoid receptor
HSP: Heat shock protein
ICS: Inhaled corticosteroids
NR3C1: Nuclear receptor subfamily 3 group C member 1

## References

[1] Agusti A, Melen E, DeMeo DL, Breyer-Kohansal R, Faner R (2022). Pathogenesis of chronic obstructive pulmonary disease: understanding the contributions of gene-environment interactions across the lifespan. Lancet Respir Med.

[2] Agusti A, Celli BR, Criner GJ, Halpin D, Anzueto A, Barnes P (2023). Global initiative for chronic obstructive lung disease 2023 report: GOLD executive summary. Eur Respir J.

[3] Barnes PJ (2000). Inhaled corticosteroids are not beneficial in chronic obstructive pulmonary disease. Am J Respir Crit Care Med.

[4] Agusti A, Hogg JC (2019). Update on the pathogenesis of chronic obstructive pulmonary disease. N Engl J Med.

[5] Wang RY, Noddings CM, Kirschke E, Myasnikov AG, Johnson JL, Agard DA (2022). Structure of Hsp90-Hsp70-Hop-GR reveals the Hsp90 client-loading mechanism. Nature.

[6] Chrousos GP (2004). The glucocorticoid receptor gene, longevity, and the complex disorders of western societies. Am J Med.

[7] Martini L, Chrousos ECTKGP (2004). Glucocorticoid receptor. Encyclopedia of Endocrine diseases.

[8] Sanchez-Vega B, Krett N, Rosen ST, Gandhi V (2006). Glucocorticoid receptor transcriptional isoforms and resistance in multiple myeloma cells. Mol Cancer Ther.

[9] Oakley RH, Jewell CM, Yudt MR, Bofetiado DM, Cidlowski JA (1999). The dominant negative activity of the human glucocorticoid receptor beta isoform. Specificity and mechanisms of action. J Biol Chem.

[10] Duma D, Cidlowski JA (2010). Generating diversity in glucocorticoid receptor signaling: mechanisms, receptor isoforms, and post-translational modifications. Horm Mol Biol Clin Investig.

[11] Li Z, Menoret A, Srivastava P (2002). Roles of heat-shock proteins in antigen presentation and cross-presentation. Curr Opin Immunol.

[12] Rosenzweig R, Nillegoda NB, Mayer MP, Bukau B (2019). The Hsp70 chaperone network. Nat Rev Mol Cell Biol.

[13] Genest O, Wickner S, Doyle SM (2019). Hsp90 and Hsp70 chaperones: collaborators in protein remodeling. J Biol Chem.

[14] Fang L, Li J, Papakonstantinou E, Karakioulaki M, Sun Q, Schumann D (2021). Secreted heat shock proteins control airway remodeling: Evidence from bronchial thermoplasty. J Allergy Clin Immunol.

[15] Papakonstantinou E, Koletsa T, Zhou L, Fang L, Roth M, Karakioulaki M (2021). Bronchial thermoplasty in asthma: an exploratory histopathological evaluation in distinct asthma endotypes/phenotypes. Respir Res.

[16] Zhou L, Fang L, Tamm M, Stolz D, Roth M (2023). Extracellular heat shock protein 70 increases the glucocorticoid receptor and dual-specificity phosphatase 1 via toll-like receptor 4 and attenuates inflammation in airway epithelial cells. Int J Mol Sci.

[17] Stolz D, Papakonstantinou E, Pascarella M, Jahn K, Siebeneichler A, Darie AM (2023). Airway smooth muscle area to predict steroid responsiveness in COPD patients receiving triple therapy (HISTORIC): a randomised, placebo-controlled, double-blind, investigator-initiated trial. Eur Respir J..

[18] Yorgancioglu A, Reddel HK, Directors GBo, Committee GS (2023). Global initiative for asthma: 30 years of promoting evidence-based asthma care. Allergy..

[19] Hogg JC, Timens W (2009). The pathology of chronic obstructive pulmonary disease. Annu Rev Pathol.

[20] Barnes PJ (2008). Immunology of asthma and chronic obstructive pulmonary disease. Nat Rev Immunol.

[21] Doeing DC, Solway J (2013). Airway smooth muscle in the pathophysiology and treatment of asthma. J Appl Physiol.

[22] Lauten M, Beger C, Gerdes K, Asgedom G, Kardinal C, Welte K (2003). Expression of heat-shock protein 90 in glucocorticoid-sensitive and -resistant childhood acute lymphoblastic leukaemia. Leukemia.

[23] Lee KH, Jeong J, Yoo CG (2013). Positive feedback regulation of heat shock protein 70 (Hsp70) is mediated through Toll-like receptor 4-PI3K/Akt-glycogen synthase kinase-3beta pathway. Exp Cell Res.

[24] Fan CS, Chen CC, Chen LL, Chua KV, Hung HC, Hsu JT (2022). Extracellular HSP90alpha induces MyD88-IRAK complex-associated IKKalpha/beta-NF-kappaB/IRF3 and JAK2/TYK2-STAT-3 signaling in macrophages for tumor-promoting M2-polarization. Cells.

[25] Lipsker D, Ziylan U, Spehner D, Proamer F, Bausinger H, Jeannin P (2002). Heat shock proteins 70 and 60 share common receptors which are expressed on human monocyte-derived but not epidermal dendritic cells. Eur J Immunol.

[26] Hornef MW, Normark BH, Vandewalle A, Normark S (2003). Intracellular recognition of lipopolysaccharide by toll-like receptor 4 in intestinal epithelial cells. J Exp Med.

[27] Wu Z, Xu Z, Zhou X, Li H, Zhao L, Lv Y (2022). sGRP78 enhances selective autophagy of monomeric TLR4 to regulate myeloid cell death. Cell Death Dis.

[28] Ferraro M, Di Vincenzo S, Dino P, Bucchieri S, Cipollina C, Gjomarkaj M (2019). Budesonide, aclidinium and formoterol in combination limit inflammaging processes in bronchial epithelial cells exposed to cigarette smoke. Exp Gerontol.

[29] Qu B, Jia Y, Liu Y, Wang H, Ren G, Wang H (2015). The detection and role of heat shock protein 70 in various nondisease conditions and disease conditions: a literature review. Cell Stress Chaperones.

[30] Cui X, Xing J, Liu Y, Zhou Y, Luo X, Zhang Z (2015). COPD and levels of Hsp70 (HSPA1A) and Hsp27 (HSPB1) in plasma and lymphocytes among coal workers: a case-control study. Cell Stress Chaperones.

[31] Hulina-Tomaskovic A, Somborac-Bacura A, Grdic Rajkovic M, Hlapcic I, Jonker MR, Heijink IH (2022). Extracellular Hsp70 modulates 16HBE cells' inflammatory responses to cigarette smoke and bacterial components lipopolysaccharide and lipoteichoic acid. Cell Stress Chaperones.

[32] Luo J, Wang Y, Dong X, Wang W, Mu Y, Sun Y (2022). miR-642a-5p increases glucocorticoid sensitivity by suppressing the TLR4 signalling pathway in THP-1 cells. Biochem Biophys Rep.

[33] Li Z, Hadlich F, Wimmers K, Murani E (2022). Glucocorticoid receptor hypersensitivity enhances inflammatory signaling and inhibits cell cycle progression in porcine PBMCs. Front Immunol.

[34] Colli LM, do Amaral FC, Torres N, de Castro M (2007). Interindividual glucocorticoid sensitivity in young healthy subjects: the role of glucocorticoid receptor alpha and beta isoforms ratio. Horm Metab Res.

[35] Dong J, Guo L, Liao Z, Zhang M, Zhang M, Wang T (2013). Increased expression of heat shock protein 70 in chronic obstructive pulmonary disease. Int Immunopharmacol.

[36] Ramos FL, Krahnke JS, Kim V (2014). Clinical issues of mucus accumulation in COPD. Int J Chron Obstruct Pulmon Dis.

[37] Raiford KL, Park J, Lin KW, Fang S, Crews AL, Adler KB (2011). Mucin granule-associated proteins in human bronchial epithelial cells: the airway goblet cell "granulome". Respir Res.

[38] Hussain SS, Edwards YJK, Libby EF, Stanford D, Byzek SA, Sin DD (2022). Comparative transcriptomics in human COPD reveals dysregulated genes uniquely expressed in ferrets. Respir Res.

[39] Wang Y, Chen T, Han C, He D, Liu H, An H (2007). Lysosome-associated small Rab GTPase Rab7b negatively regulates TLR4 signaling in macrophages by promoting lysosomal degradation of TLR4. Blood.

[40] Heyl KA, Klassert TE, Heinrich A, Muller MM, Klaile E, Dienemann H (2014). Dectin-1 is expressed in human lung and mediates the proinflammatory immune response to nontypeable Haemophilus influenzae. mBio.

[41] Safi S, Messner L, Kliebisch M, Eggert L, Ceylangil C, Lennartz P (2023). Circulating Hsp70 levels and the immunophenotype of peripheral blood lymphocytes as potential biomarkers for advanced lung cancer and therapy failure after surgery. Biomolecules.

[42] Liang Z, Wang F, Zhang D, Long F, Yang Y, Gu W (2020). Sputum and serum autoantibody profiles and their clinical correlation patterns in COPD patients with and without eosinophilic airway inflammation. J Thorac Dis.

[43] Liu J, Jiang Y, Jiang Z, Feng Y, Zhao R (2022). Distinct patterns of GR transcriptional regulation in liver and muscle of LPS-challenged weaning piglets. Int J Mol Sci.

