# Supplementary material for: Heat-Induced Secretion of Heat Shock Proteins 70 and 90 Does not Affect the Expression of the Glucocorticoid Receptor in Primary Airway Cells in COPD
Source: Lung. 2024 Apr 19;202(3):235–43. doi: 10.1007/s00408-024-00680-8 (PMC11143057; doi:10.1007/s00408-024-00680-8)
Supplement: Supplementary file 1 — Supplementary file1 (DOCX 585 kb) [file 408_2024_680_MOESM1_ESM.docx]

**Appendix**


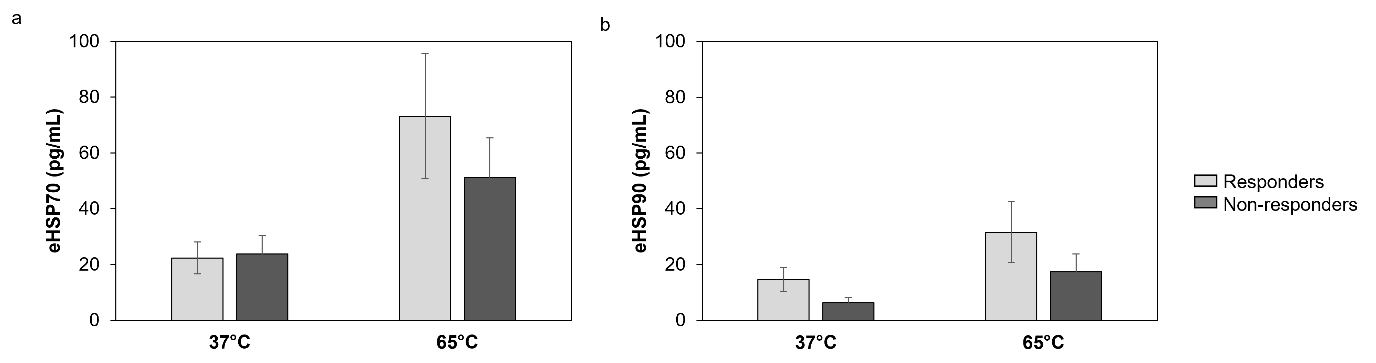


**Supplementary Fig. 1** a: eHSP70 secretion from primary COPD BEC (n=23) before and after heat exposure (10 second 65^o^C) was measured by ELISA. Among these 23 patients, 7 patients responded to ICS and 16 patients did not. b: eHSP90 secretion from primary COPD BEC (n=23) before and after heat exposure (10 second 65^o^C) was measured by ELISA. Among these 23 patients, 7 patients responded to ICS and 16 patients did not. Bars show mean±S.E.M. P-values were calculated by Student’s t-test and revealed no significant difference (not shown)


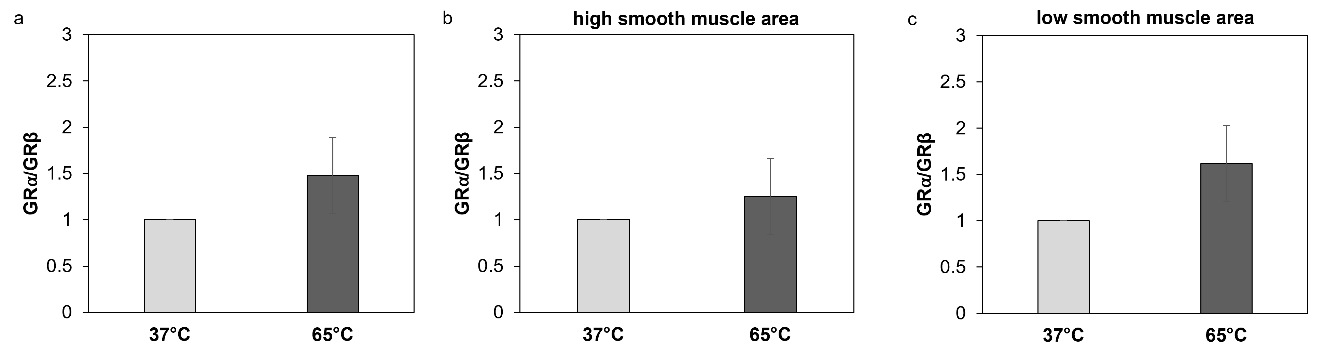


**Supplementary Fig. 2** a: Ratio of GRα to GRβ in COPD BEC (n=16) exposed to heat (10 second 65^o^C). Βars represent fold change of ratio of GRα to GRβ to cells cultured under 37^o^C. b-c: Among these 16 patients, 6 patients had high smooth muscle mass, and 10 patients had low smooth muscle mass. Bars present fold change of ratio of GRα to GRβ to cells cultured under 37^o^C, and show mean±S.E.M. P-values were calculated by Student’s t-test and revealed no significant difference (not shown)


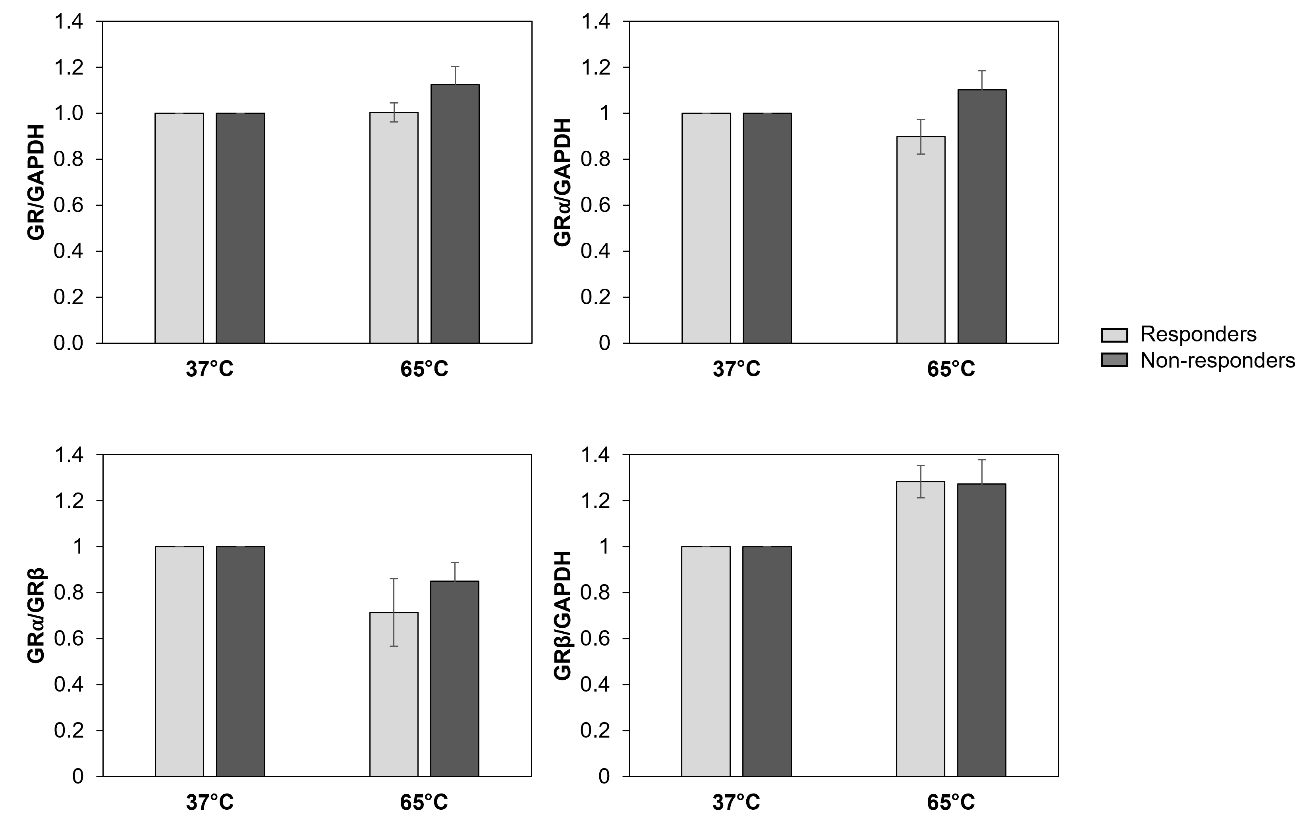


**Supplementary Fig. 3** GR and GR-isoforms expression in primary BEC from COPD patients (n=16) exposed to heat treatment. Among these 16 patients, 6 patients responded to ICS and 10 patients did not. Βars represent fold change of GR, GRα and GRβ to cells cultured at 37^o^C. Expression of total GR represents the sum of the expression of GRα and GRβ isoforms. Bar shows mean±S.E.M. P-values were calculated by Student’s t-test and revealed no significant difference (not shown)


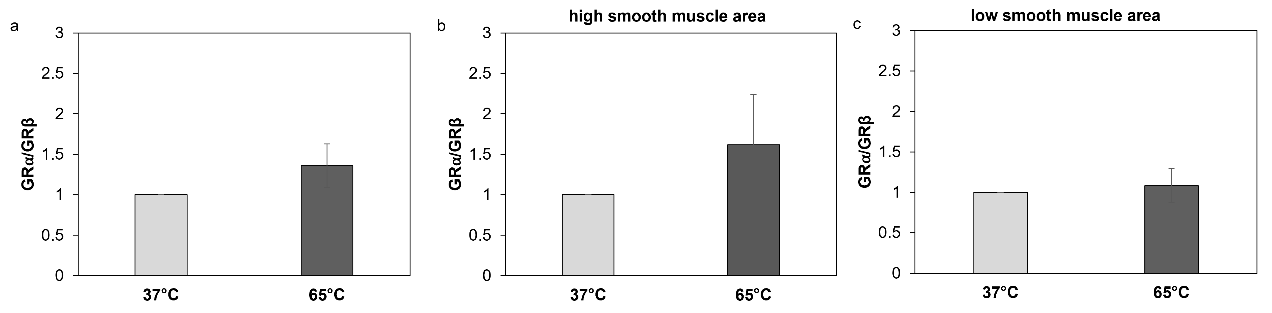


**Supplementary Fig. 4** Ratio of the expression of GRα to GRβ in COPD ASMC (n=16) exposed to heat. Βars represent fold change of ratio of GRα to GRβ to cells cultured under 37^o^C. b-c: Among these 16 patients, 6 patients had high smooth muscle mass, and 10 patients had low smooth muscle mass. Bar present fold change of ratio of GRα to GRβ to cells cultured at 37^o^C, and show mean±S.E.M. P-values were calculated by Student’s t-test and revealed no significant difference (not shown)


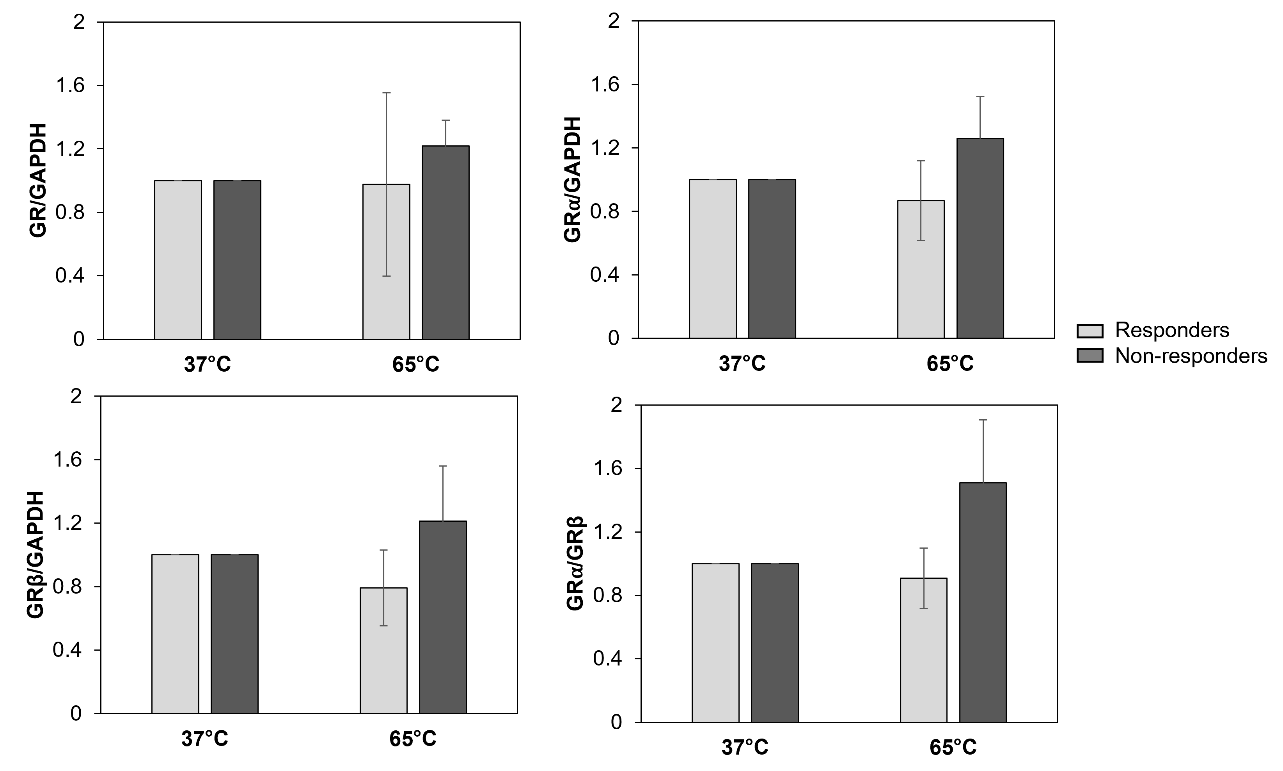


**Supplementary Fig. 5** GR and GR-isoforms expression in primary ASMC from COPD patients (n=15) exposed to heat treatment. Among these 15 patients, 6 patients responded to ICS and 9 patients did not. Βars represent fold change of GR, GRα and GRβ to cells cultured at 37^o^C. Expression of total GR represents the sum of the expression of GRα and GRβ isoforms. Bars show mean±S.E.M. P-values were calculated by Student’s t-test and revealed no significant difference (not shown)

**
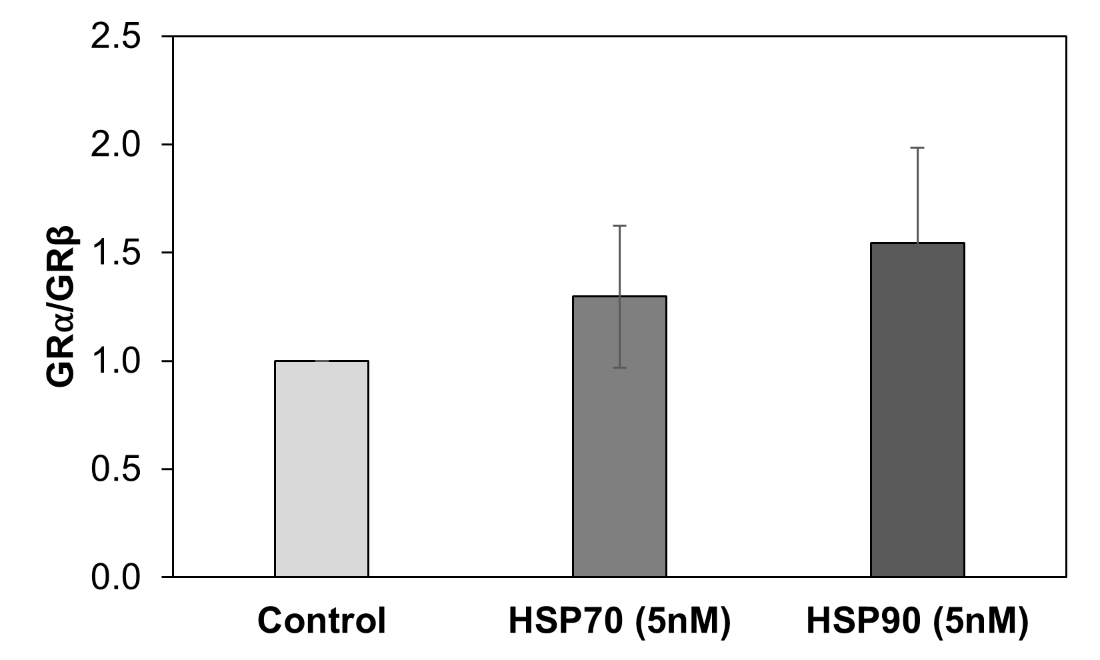
**

**Supplementary Fig. 6** Ratio of GRα to GRβ in COPD ASMC (n=7) exposed to HSP70 (5nM) or HSP90 (5nM) over 24 hours. Bars present fold change of ratio of GRα to GRβ to cells without eHSPs treatment, and show mean±S.E.M. P-values were calculated by Student’s t-test and revealed no significant difference (not shown)
